# lncRNA PVT1 in the Pathogenesis and Clinical Management of Renal Cell Carcinoma

**DOI:** 10.3390/biom11050664

**Published:** 2021-04-29

**Authors:** Julia Bohosova, Adela Kubickova, Ondrej Slaby

**Affiliations:** 1 Central European Institute of Technology, Masaryk University, 625 00 Brno, Czech Republic; julia.bohosova@ceitec.muni.cz; 2 Department of Pharmacology, Faculty of Medicine, Masaryk University, 625 00 Brno, Czech Republic; adela.kubickova@med.muni.cz; 3 Department of Biology, Faculty of Medicine, Masaryk University, 625 00 Brno, Czech Republic

**Keywords:** biomarker, prognosis, diagnosis, long non-coding RNA, tumorigenesis

## Abstract

LncRNA PVT1 (plasmacytoma variant translocation 1) has become a staple of the lncRNA profile in patients with renal cell carcinoma (RCC). Common dysregulation in renal tumors outlines the essential role of PVT1 in the development of RCC. There is already a plethora of publications trying to uncover the cellular mechanisms of PVT1-mediated regulation and its potential exploitation in management of RCC. In this review, we summarize the literature focused on PVT1 in RCC and aim to synthesize the current knowledge on its role in the cells of the kidney. Further, we provide an overview of the lncRNA profiling studies that have identified a more or less significant association of PVT1 with the clinical behavior of RCC. Based on our search, we analyzed the 17 scientific papers discussed in this review that provide robust support for the indispensable role of PVT1 in RCC development and future personalized therapy.

## 1. Introduction

Tumors of the kidney (renal cell carcinoma, RCC) account for 2% of all cancers [1]. Although mortality is slowly decreasing [2], RCC is considered afatal disease, with a relatively high percentage of patients having developed metastasis at the time of the diagnosis and with low chances for 5-year survival [2]. The exact identification of the RCC entity or subtype is necessary for accurate and effective treatment and proper prognosis assessment. The WHO classification system divides RCC into sixteen subtypes, the most common being three of them—clear cell carcinoma (ccRCC) with 75% abundance of all RCC cases, followed by papillary renal cell carcinoma (PRCC; 15–20%) and chromophobe renal cell carcinoma (chRCC; 5%) [3]. CcRCC is also the most aggressive type of renal cancer with the worst prognosis than the less-frequent subtypes [3,4].

Prognosis is assessed based on the TNM classification system (T—the size of the primary tumor, N—the presence of the metastases in regional lymph nodes, and M—distant metastases positivity) describing the tumor’s stage. Other prognostic information is brought in by the histologic type and level of the tumor’s differentiation [5]. From a diagnostic and prognostic point of view, it is possible to discriminate between individual RCC subtypes using immunohistochemistry or using gene expression panels [3]. With the development of targeted therapy, more attention has been turned to predictive biomarkers [6]. However, there is currently no biomarker reaching a sufficient specificity and sensitivity enabling the precise personalization of the therapy [5], although great scientific effort has been focused on searching for biomarkers in many human diseases [7], including RCC [5]. Among the potential molecules feasible as biomarkers, long non-coding RNAs have been studied excessively. Many studies emerged, mainly after expansion of next-generation sequencing, focusing on a lncRNA as diagnostic, prognostic, or predictive biomarkers.

In 2008, one of the first studies described the dysregulation of lncRNA expression levels in renal cell carcinoma. The authors observed deregulated levels of seven intronic lncRNAs in tumor samples compared to nontumor renal tissue [8]. Subsequent microarray profiling showed thousands of lncRNAs with differential expression in RCC tumor tissue [9]. Since then, *PVT1* has repeatedly emerged in many profiling studies as a prominently dysregulated lncRNA in renal cell carcinoma, even in comparison with other human tumors [10]. Therefore, we decided to summarize the current knowledge on this lncRNA and its role in the development and progression of RCC, with a particular focus on its potential feasibility as a diagnostic and prognostic biomarker. To review the literature, we searched the PubMed database for relevant studies published from inception to 23 April 2021. Using search terms (“PVT1” and “renal” “and “cell” and “carcinoma”) and excluding studies that were not primarily focused on renal cell carcinoma patients or working with non-RCC cell lines, we identified 17 articles relevant for further discussion. The papers focused on lncRNA profiling, containing biomarker discovery, or the analysis of profiling data as a part of study design are summarized in Table 1. The papers focused on *PVT1* in a cellular context or containing an in vitro functional test as a part of the study design are discussed in the subsequent section.

## 2. General Characterization of Long Non-Coding RNAs

Sequencing of the human genome revealed that the number of protein-coding genes reaches up to 20,000 to 25,000 [11], which makes up around 1.5% of the human genome. The remaining part of the genome serves structural or regulatory functions. Depending on the cell type, up to 85% of the genome is actively transcribed and gives rise to many different types of untranslated transcripts [12,13]. These transcripts were termed non-coding RNAs [14], and new knowledge about their wide range of functions in the human body has emerged in recent decades. The database NONCODE currently states that lncRNAs originate from 96,000 genes producing more than 172,000 transcripts [15]. LncRNAs occur in different tissues, and in contrast to mRNAs, their temporospatial specificity is higher, even though their expression levels usually remain low [16,17,18,19]. They are localized preferably in the cell nucleus, close to chromatin, but can also be found in the cytoplasm [13,19]. According to an extensive analysis of the translational potential of annotated lncRNAs, most of them should not code a functional protein [20], although some rare exceptions have been observed [21,22].

The genomic structure of lncRNAs is similar to mRNA originating from protein-coding genes [19]. LncRNAs are transcribed by RNA polymerase II, usually contain a 5’cap, and are polyadenylated at the 3’ end. However, there are some exceptions [23,24], and several alternative lncRNA-editing pathways have been described [25]. In 98% of all cases, lncRNAs are spliced and usually contain only two exons, much less than in protein-coding mRNA. In more than 25% of all lncRNA transcripts, splicing creates several isoforms [18,19]. Interestingly, lncRNAs can be a source or a precursor of sncRNAs, such as tRNA, miRNA, snRNA, and above all, snoRNA [13,19].

The function can be predicted from the sequence and level of conservation, and because most lncRNAs are less conserved than protein-coding genes, they have long been considered nonfunctional [26,27]. However, the conservation of lncRNAs is higher than those of introns or intergenic regions. Moreover, the promotors of lncRNAs are conserved comparably to the promotors of protein-coding genes [19,23,28,29,30,31]. Taken together with a wide and omnigenous palette of means by which lncRNAs are involved in the life of the cell, their great evolutionary importance is evident [32,33].

LncRNAs regulate cellular gene expression using various mechanisms on the transcriptional, posttranscriptional, and translational level. There are four main archetypes or mechanisms of regulation. LncRNAs function as molecular signals when their presence serves as an indicator of the transcriptional activity, because their expression is time-, stimuli-, or site-specific. Another type is a molecular decoy when lncRNAs bind to other regulatory molecules, such as transcription factors or chromatin remodeling complexes, and lower their availability. Moreover, lncRNAs serve as a guide, interacting with proteins creating ribonucleoprotein complexes guided by lncRNA to specific targets, usually to nearby (*cis*) or distant (*trans*) genes. A necessary function is a scaffold, where lncRNA functions as a platform for assembling different molecular components, creating RNP complexes that can activate or inhibit transcription or have a structurally stabilizing effect [34].

The mechanisms of regulation of the gene expression by lncRNAs are inevitably joined with their localization in a cell, which determines what structures or processes will be affected [19]. Nuclear lncRNAs regulate the gene expression on three levels—they affect the chromatin and transcription and posttranscriptional modifications of mRNA. Nuclear lncRNAs can accumulate in *cis* (close to the side of their own transcription) or in *trans* (being dislocated from the site of their origin to fulfil their function somewhere else). However, lncRNAs accumulated in *cis* can also act in *trans* [35].

An important function of lncRNA is their involvement in epigenetic regulation—specifically, chromatin remodeling. LncRNAs bind to chromatin modification factors such as methyltransferases [36,37] and guide them to the specific loci in chromatin or, on the other hand, prevent them from binding to the chromatin [38,39].

The second level is transcriptional regulation and its activation or repression by three different mechanisms concerning lncRNAs [40]. Firstly, lncRNAs bind activating proteins and protein complexes [41], including transcription factors [42]. Further, they mediate chromatin interactions, including chromosome looping, which enables the interactions of genes and regulatory elements originating from the distant loci on the chromosome [43,44]. LncRNAs also prevent repressive mechanisms from action [45,46] or, conversely, can act as corepressors of transcription [47,48]. On a post-transcriptional level, lncRNAs cofacilitate pre-mRNA splicing [49,50].

In a cytoplasm, lncRNAs regulate the gene expression on a post-transcriptional and post-translational level, influencing the mRNA stability in a positive [51] or negative [52] way. LncRNAs affect even the translation of mRNA itself, both as a suppressor [53] and as an activator [54].

A separate category of lncRNAs is a network of so-called competing endogenous RNAs (ceRNAs), which function as microRNA (miRNA) sponges binding miRNAs and averting them from binding to their target mRNAs [55].

LncRNAs can bind proteins and prevent them from the transcriptional regulation of target genes [56] or maintaining genomic stability [57]. Protein stability and turnover can be affected by the lncRNAs involved in ubiquitin-mediated proteolysis [58]. Conversely, some lncRNAs can prevent proteins from ubiquitination [59]. It has been described that lncRNAs are involved in signaling pathways and the cellular transport of molecules, including other lncRNAs [60], which may function in organelles [61].

As lncRNAs are crucial components of many cellular processes and their functional scope is broad, it is evident that their dysregulation could lead to pathological conditions and the development of various diseases, depending on the mechanism that was disrupted. LncRNAs are associated with neurological and neurodegenerative diseases [62], such as Alzheimer’s disease [63], diabetes mellitus and its complications [64], autoimmune [65], inflammatory [66], or cardiovascular diseases [67]. The most attention, however, has been given to tumor diseases. LncRNAs can act both as oncogenes and tumor suppressors. They regulate the expression of such genes [68] and modulate all the tumor hallmarks, according to Hanahan and Weinberg [69], such as the independent production of proliferation signals, insensitivity to antigrowth signals and programmed cell death escape [70,71], genomic instability, inflammation, disruption of energetic metabolism, and immune surveillance escape [72].

## 3. Role of PVT1 in Development of Renal Cell Carcinoma

Encoded by the human *PVT1* (plasmacytoma variant translocation 1) gene of nine exons, *PVT1* is 1957-bp-long lncRNA located at 8q24.21 [73]. *PVT1* is generally known as an oncogene involved in tumorigenesis [74]. The artificial silencing of *PVT1* represses EMT and affects the proliferation, apoptosis, migration, and cell cycle by regulating many prominent tumorigenesis players, such as cyclin D1, p21, and Myelocytomatosis (MYC) [75,76,77,78]. The latter mentioned originates in a famous proto-oncogene MYC localized close to the *PVT1* gene. *PVT1* and *MYC* are known to interact with each other and are commonly coamplified [79].

**Figure 1:**
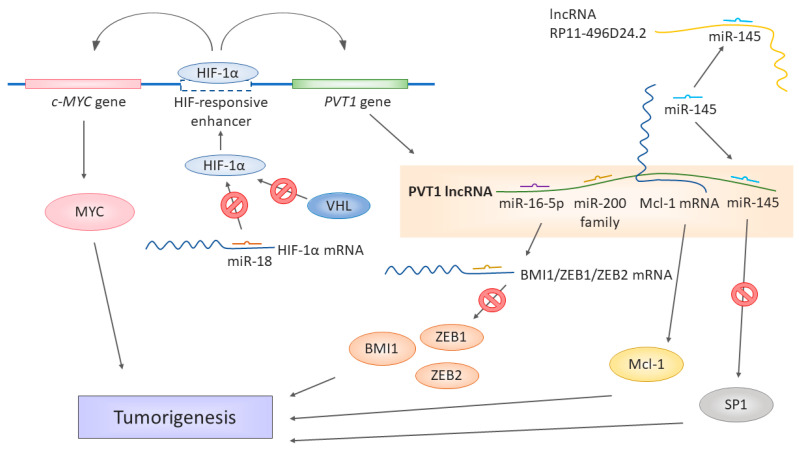
Different ways of lncRNA *PVT1* contributing to the development of renal cell carcinoma. miR-18 binds to *HIF-1α* mRNA and prevents it from translation. If downregulated, the miR-18 levels are not sufficient to keep the expression of *HIF-1α* in check. However, while the normal oxygen levels last, protein HIF-1α is targeted by VHL for degradation. In hypoxia, HIF-1α is stabilized and cannot be targeted by VHL and can be transferred to the nucleus. The *PVT1* gene is under the HIF-responsive enhancer, along with *cMYC*. HIF-1α binding to this enhancer activates the expression of both *c-MYC* and *PVT1*. *PVT1* itself serves as a miRNA sponge binding the miR-16-5p, miR-145, and miR-200 family members, thus liberating the target mRNA from miRNA-induced degradation, for example, *BMI1, ZEB1, ZEB2*, and *SP1*. miR-145 is a target of both *PVT1* and *RP11-496D24.2* in the ceRNA network. Moreover, *PVT1* stabilizes larger mRNA molecules such as *Mcl-1*, which, in turn, can be translated into functional Mcl-1 proteins. The upregulation of *PVT1* is thus associated or directly responsible for the upregulation of significant factors promoting tumorigeneses, such as MYC, Mcl-1, BMI1, ZEB1, ZEB2, and SP1.

Corresponding with the dysregulation in RCC tumor samples, *PVT1* is overexpressed in RCC cells as well. The results show that *PVT1* affects apoptosis through myeloid leukemia cell differentiation protein 1 (Mcl-1), a member of the B-cell leukemia lymphoma protein (Bcl-2) family of proteins involved in regulating cell death and comprising both pro- and antiapoptotic factors. Mcl-1 is antiapoptotic; thus, its upregulation leads to cell death signal resistance and increased survival typical for human cancer cells [80]. *PVT1* knockdown in RCC cells leads to the downregulation of Mcl-1 as *PVT1* enhances the *Mcl-1* mRNA stability, thus keeping its levels higher (Figure 1). Downregulating either member of the *PVT1*/Mcl-1 axis inhibits proliferation and colony formation and promotes apoptosis [81].

*PVT1* seems to be also tightly connected to VHL signaling and hypoxia sensing [82], a significant feature of RCC tumorigenesis. On a molecular level, the most frequent cause of either sporadic or hereditary RCC is the inactivation of the von Hippel-Lindau (*VHL)* gene [83]. Mutations causing the loss of heterozygosity of the 3p chromosome [83,84,85] or hypermethylation of the *VHL* promotor [86] are common in RCC patients. Defective protein VHL enables the stabilization of the hypoxia-induced factor (HIF) protein family, which would be otherwise degraded in a proteasome. When stabilized, the HIF protein family activates and regulates the cellular response to hypoxia, affecting the genes involved in angiogenesis (for example, *VEGF*—vascular endothelial growth factor), cell migration, or apoptosis. VHL thus functions as a tumor suppressor [87,88].

The results of Grampp et al. [89] tie the involvement of *PVT1* in MYC and VHL regulation together (Figure 1), as their team identified a unique polymorphism at 8q24.21 associated with renal cancer susceptibility. The polymorphism influences the expression of MYC and *PVT1*, as it is localized in the HIF-binding enhancer, affecting both the cellular MYC (*c-MYC)* gene and *PVT1* gene. The polymorphism’s effect is restricted to renal tubular cells, and the renal cancer susceptibility depends on the genotype of the respective polymorphism site in the enhancer, affecting its accessibility to HIF proteins. pVHL defects enhanced expression of c-*MYC* and *PVT1*, hence the notorious upregulation of *PVT1* in RCC. The polymorphisms in the HIF-responding regulatory elements could affect renal tumorigenesis. As the authors pointed out, the currently known SNPs associated with RCC are all linked to modulation of the VHL/HIF pathway [89,90]. Therefore, the renal cancer-promoting or protective effect of a given SNP depends on its effect on the HIF expression and HIF-mediated regulation [89].

The regulation of oxygen sensing has been expanded for the role of miR-18a [82], which regulates the expression of the HIF-1α protein. The downregulation of miR-18a leads to higher levels of the HIF-1α protein, whose expression is then unrestricted and affects the expression of *PVT1* (Figure 1). The miR-18a/HIF-1α/*PVT1* regulatory pathway plays a crucial role in the development and prognosis of ccRCC, as miR-18a has been identified in this work as a significant biomarker, and prognosis and RCC development [82].

The complexity of gene expression regulation by non-coding RNAs was further illustrated by Yang et al. [73] in their work showing that *PVT1* also serves as a ceRNA in the context of RCC. The *PVT1* levels negatively correlate with the miR-200 family and miR-20a, miR-20b, and miR-203s. *PVT1* shares the miRNA-binding “seed” sequence with the miR-200-regulated mRNAs of *BMI1, ZEB1*, and *ZEB2*, all three being major players in cancer [91,92,93] (Figure 1). The subsequent in vitro experiments showed that *PVT1* upregulates the levels of BMI1, ZEB1, and ZEB2, while the overexpression of the miR-200s downregulated them. The authors further identified a new splicing variant of *PVT1*, whose levels were even higher in the tumor tissue and tumor cell lines than the levels of the full-length original splicing variant and had an even higher effect on the cell proliferation. The spliced region, exon 4, does not contain binding sites for the miR-200 family. Therefore, even this variant can act as ceRNA [73]. Ren et al. [77] identified the functional connection of *PVT1* with miR-16-5p, as the silencing of this miRNA has led to the reversion of the regulatory effect on RCC cells induced by the downregulation of *PVT1*. An in-silico target prediction showed that miR-16-5p is a target of *PVT1*. Thus, its levels decrease as a result of binding to *PVT1* (Figure 1). Moreover, the strong negative regulatory connection of *PVT1* and miR-16-5p has been observed in colorectal cancer [94].

A similar ceRNA mechanism has been observed in PRCC by Huang et al. [95]. In their work, they found the interactions of miR-145 miR-211, miR-216a, mIR-133a, and miR-133b with *PVT1*. These interactions overlapped with lncRNA *RP11-496D24.2*, which also contained binding sites for some of these miRNAs (mir-145, mir-211, and mir-216a). The authors suggested a competition mechanism between *PVT1* and *RP11-496D24.2* for these common miRNAs, thus being critical regulators of pathogenesis in PRCC. However, the downstream targets profiting from *RP11-496D24.2*-mediated miRNA binding are not yet known. In *PVT1*, though, SP1 has been identified as a potential target of *PVT1*, miR-145, miR-133a, and miR-133b. SP1 is a versatile transcription factor [96] involved in many cellular processes as an activator or repressor, depending on the context. Suggested lncRNA-miRNA-mRNA ceRNA pathway regulating of such a potent regulator of gene expressions could be a significant driving force in the pathogenesis of PRCC, especially with the upregulation of *PVT1*, which was shown by Huang et al. [95] in PRCC patients.

The complex system of *PVT1* tumorigenesis regulation via the regulation of miRNA was exceptionally summarized in the work of Wang et al. [97]. PVT1 functions as a miRNA “sponge”, inhibiting their activity by lowering the levels of more than 20 miRNAs. Should those miRNAs be involved in the regulation of other oncogenic mRNAs, the upregulation of *PVT1* would lead to their decrease and, thus, the promotion of tumorigenesis. Moreover, *PVT1* seems to be a precursor to other miRNAs with either oncogenic or tumor-suppressive effects [97].

## 4. *PVT1* as a Diagnostic and Prognostic Biomarker

The prominent association of *PVT1* with renal cell carcinoma has been outlined repeatedly in lncRNA profiling studies (Table 1). Posa et al. [98] analyzed over 700 samples of patients with different types of cancer, including RCC. Already here, the upregulation of *PVT1* in RCC overshadows other types of tumors. This trend is promising for further uses in diagnostics, and luckily, subsequent papers seem to validate *PVT1* as a specific marker of ccRCC. However, *PVT1* was shown to also be significantly upregulated in PRCC [95]. Liu et al. [99] later identified a panel of four lncRNAs, including *PVT1*, specific for ccRCC, and developed a risk score for the ccRCC prognosis estimation. Wang et al. [100] created a model of independent prognostic lncRNAs, miRNAs, and mRNAs, including *PVT1*, associated with the overall survival. Moreover, lncRNAs *PVT1*, *LINC00472, TCL6, WT-AS1*, and mRNA of the COL4A4 protein were identified as independent prognostic factors.

Interestingly, the association with the tumor stage seemed to be highest in stage III, while in stages I, II, and IV, there were lower proportions of patients with a high level of *PVT1* [98]. Conversely, Wu et al. [81] used TCGA data as well; however, they observed an increase in the *PVT1* expression with the histological and TNM stages.

The association of *PVT1* upregulation with several clinicopathological parameters is popping up repeatedly in several works. The most prominent is the association with the TNM stage [73,77,81,103]. Li et al. [75] identified the association of *PVT1* expression with the T, M, and AJCC stages but not the N stage and grade. Other works have frequently identified the association of *PVT1* expression with the histological grade, Fuhrman stage, distant metastasis, overall survival, and disease-free survival, and the results typically overlap. In almost all of the reviewed publications, *PVT1* upregulation correlates with a shorter survival in RCC patients, even though some exceptions need to be highlighted [102] where *PVT1* was not among the most prominent candidates. Although this would seem promising, most of the papers utilized the same TCGA cohorts repeatedly; only some also recruited independent cohorts of RCC patients [73,75,81,89,99]. Typically, the TCGA cohort was used for exploration, while the patient samples served as a validation cohort. Only three papers utilized independently collected patient samples [77,101,103]. An independent cohort showed its importance for Wu et al. [81], who observed differences in the TCGA and independent cohorts, as *PVT1* was more upregulated in the TCGA one.

Possibly, the highest aim for biomarker studies is to find a molecule feasible in so-called liquid biopsy— biomarkers detectable in body fluids with little to no burden for the patient, faster and simpler analyses, and precise results. The potential use of lncRNA in the form of liquid biopsy from bodily fluids stems from their stability, although the exact mechanism is still unclear [101]. The encapsulation of lncRNAs in vesicles such as apoptotic bodies, microvesicles, and exosomes or the formation of complexes with proteins might play a part [104,105,106,107]. So far, the only study aimed in this direction was provided by Wu et al. [101]. The authors measured 82 lncRNAs chosen from the literature in the tissues of ccRCC, chromophobe RCC, and PRCC.

Further validation in the tissues and serum of ccRCC patients and healthy controls led to a five lncRNA diagnostic model. Combining *lncRNA-LET, PVT1, PANDAR, PTENP1*, and *linc00963* provided a diagnostic ability with the area under the curve of 0.90. Interestingly, *PVT1* was downregulated in the serum ccRCC samples (*p* = 0.021), which did not correspond with the higher expression in the ccRCC tissues. The authors suggested that this discrepancy might be caused by the mechanism of lncRNA export from the cell. The recruitment of lncRNA into extracellular vesicles can be facilitated through ECV-bound proteins, limiting the capacity of transferable lncRNAs. Therefore, it might not reflect the levels in the tissues [101,108,109].

## 5. Conclusions

Many new lncRNAs have been described and studied in recent years thanks to the extensive development of sequencing technology and bioinformatics approaches primarily for data assessment. A quick search in PubMed returned more than 400 papers on lncRNAs in renal cell carcinoma. *PVT1* has become a staple of the standard RCC lncRNA profile, as it has been repeatedly identified among the most dysregulated lncRNAs in RCC. Its role in regulating the cellular processes has been investigated in several papers, which show that *PVT1* is a significant factor promoting tumorigenesis. Association with the development of a tumor creates a biomarker potential, which can be put to good use. Dysregulation of the *PVT1* expression is easily detectable and correlates with a specific clinical behavior and RCC subtype.

Although so far, most of the studies used TCGA datasets, several groups also added their independent exploratory cohorts. However, more alarming is the lack of independent validation by qPCR, which belongs to the gold standard of study design when searching for potential diagnostic, prognostic, and predictive biomarkers. Stemming from the use of TCGA datasets, most of the studies were focused solely on ccRCC. Only two [69,82] also included other types of renal cancer, which supports the results in the context of day-to-day diagnostic conditions and expectations from a potential biomarker. The ability to differentiate ccRCC from PRCC, chRCC, and other rarer types is more than desirable.

Nevertheless, *PVT1* showed a specific upregulation in ccRCC, which outlined its future use in clinical practices. Moreover, *PVT1* seems to be a good marker of the worse prognosis and shorter survival of patients with higher *PVT1* levels. Either singularly or in combination with other non-coding RNAs, *PVT1* should have its place in therapy personalization in the future for its fundamental biomarker value and the undeniable role in RCC development.

## Figures and Tables

**Table 1:** *PVT1* in lncRNA profiling studies in renal cell carcinoma. *—Papillary renal cell carcinoma; otherwise, clear cell renal cell carcinoma; **—Included patients with ccRCC, chromophobe RCC, and PRCC; and ***—Included both serum and tissue samples; otherwise, only tumor and nontumor tissues.

Reference	Primary Endpoint	*N* of Patients/Controls (Source)	Diagnostic Properties	Prognostic Properties	
			*p*-Value, AUC	*p*-Value	Risk Ratio, 95% CI
Studies focused on the diagnostic properties of PVT1 only					
Wu et al., 2016 ** [101]	-	71/62 healthy + 8 benign (patient samples) ***	0.007, 0.900	-	-
Grampp et al., 2016 ** [89]	-	453/453 (patient samples) 138/138 (TCGA)	<0.05	-	-
Studies focused on the prognostic properties of PVT1 only					
Posa et al., 2016 [98]	OS	-/- (TCGA)	-	<0.0001	-
Huang et al., 2017 * [95]	OS	289/32 (TCGA)	-	0.024 (panel of 15 lncRNAs)	-
Wu et al., 2017 [81]	OS	55/55 (patient samples) 534/72 (TCGA)	-	0.00007	-
Xu et al., 2017 [102]	OS	530/72 (TCGA) 109/100 (microarray)	-	9.15 × 10^−^^7^	-
Wang et al., 2018 [100]	OS	539/72 (TCGA)	-	0.0002 (panel of 11 lncRNAs)	1.47, -
Liu et al., 2020 [99]	OS	525/- (TCGA) 60/60 (patient samples)	-	00002	1.79, 1.32–2.43
Studies showing both the diagnostic and prognostic properties of PVT1					
Yang et al., 2017 [73]	OS DFS	50/50 (patient samples), 534/72 (TCGA)	<0.001	0.014 NS	1.494, 1.081–2.063 1.469, 0.976–2.211
Bao et al., 2018 [103]	OS DFS	129/129 (patient samples)	<0.01	0.012 0.004	4.445, 1.515–8.392 3.553, -
Li et al., 2018 [75]	OS	448/67 (TCGA) 40/40 (patient samples)	<0.001	<0.01	-
Ren et al., 2019 [77]	OS	25/25 (patient samples)	<0.001	0.007	-
